# Beyond the Numbers: Describing Care at the End of Life

**DOI:** 10.1371/journal.pmed.1001181

**Published:** 2012-02-28

**Authors:** The PLoS Medicine Editors

## Abstract

In the February editorial, the *PLoS Medicine* editors reflect on recent research describing care for cancer patients in the final days of life.

One of the great achievements of modern science and technology is that life expectancy has increased considerably [1]. While we cannot ignore the steep regional disparities that exist across the globe [2], for many individuals diseases that were previously life threatening are now easily remedied or no longer encountered. Still, it is inevitable that our bodies will eventually fail and, although many in the industrialized world can hope for a good quality of life, the question remains: can we be sure of a good quality of death?

In an article recently published in *PLoS Medicine*, Olav Lindqvist and colleagues tackled an emotive and often taboo subject: caregiving for the dying [3]. For the researchers, a desire to focus on the nonpharmacological aspects of care for cancer patients in the last days of life presented unique and important challenges. Conducting unobtrusive research in this most sensitive of times is not straightforward or easy; and picking apart the complexities of care giving does not easily lend itself to conventional quantitative methods of health research. However, the aim of this study was not to determine the best approach or to quantify how much is done at the end of life. Rather the authors wanted to explore a simple question that has not so far been examined in depth: what is the range of nonpharmacological care used by caregivers in the last days of life?

Lindqvist and colleagues explored the types of nonpharmacological caregiving activities that were reported by staff from 16 palliative care facilities in Argentina, New Zealand, and seven European countries. The researchers used a “freelisting approach” where exhaustive lists of responses to a question are generated by a group of interest in order to capture the full scope of answers relevant to the group. In this case, Lindqvist and colleagues asked care staff to describe the interventions and activities they carried out with patients and families during the last days of life. Over 900 statements, which were initially generated during discussions and then progressively generated as staff continued to interact with patients and their families, formed the basis of the analysis. By categorizing the character of the activities described in the statements and defining to whom care was directed, Lindqvist and colleagues were able to identify common themes and explore the interrelationships between caregiving activities. The results are complex, because the described activities often encompassed a wide range of purposes and intentions. However, an underlying theme is that efforts are made by palliative care staff to personalize care and support links with a dying individual's everyday life.

The care for patients was found to center on both physical and emotional comfort with bodily care, and contact was often highlighted in the collected statements. The contact between caregiver and patient ranged from attending to a physical need, for example, keeping a patient's lips and tongue moist, to providing emotional comfort, such as holding a patient's hand. There was also an emphasis by care staff on creating a pleasing environment for a patient. Small acts such as keeping a patient's slippers by their bed even though they are unable to walk demonstrate the subtle ways in which caregivers provide comfort by maintaining a connection with a patient's life.

One of the more potent insights reported by Lindqvist and colleagues was the tension they observed between intervening and abstaining by caregivers. For example, staff sometimes had to choose between maintaining a quiet and comfortable atmosphere and providing a patient with information. Reported quotes such as “*dare to be silent with the dying person*” reveal that it can be difficult for caregivers to overcome an emotional drive to act in response to a person who is dying.

The study also revealed that, near the end of life, caregiving by staff extended beyond the patient to include families, with care that ranged from rituals, both spiritual and legal, to practical considerations, such as removing a wheelchair from a family's house once a person had died. The activities reported in the study were often interpreted as trying to help the family come to terms with death and take leave of the person who has died; in some instances this was as simple as allowing family members to be with the body after death. The analysis suggests that caregivers recognize the process of dying as being neither focused on one fixed time point nor concerned with only the dying individual.

The qualitative approach taken by the researchers enabled the complex and often subtle behavior and intentions of palliative care staff to be explored. While this insight is important and provides a baseline on which to build other studies, the findings do not provide an understanding of the extent to which patients or their family benefit from the caregiving activities provided by staff. Further research into the last days of life will continue to require innovative methods and the use of appropriate measures, such as observed pain or discomfort [4], to probe how patients' final days can be improved.

Unfortunately, portrayals of care for the dying in the media can be dominated by anecdote, often negative [5], and other emotive issues, such as assisted dying [6]. However, the findings from this study should be considered a positive news story. They reveal the complex and sometimes subtle caregiving approaches that palliative care staff take to improve the experience of dying for both patient and family. The findings also reaffirm definitions of palliative care, which state that the scope of palliative care is not wholly centered on relief of pain and suffering but also extends to psychosocial and spiritual care [7]. Research such as this not only provides hope that it is possible to have a good quality of death but also suggests that through research the experience of dying can be improved.
